# Supplementary material for: Vibrational Spectroscopy in Urine Samples as a Medical Tool: Review and Overview on the Current State-of-the-Art
Source: Diagnostics (Basel). 2022 Dec 22;13(1):27. doi: 10.3390/diagnostics13010027 (PMC9818072; doi:10.3390/diagnostics13010027)
Supplement: Supplementary file 1 [file diagnostics-13-00027-s001.zip › Supplemental material-Table S1 - Papers' Timeline.pdf]

Table S1: Supplementary Materials. Papers' Timeline; F.Vigo et al., 2022

	I. Autor	Year	Title	Type	Technik	Statistical Algorithms	Statistical Software	Enhancing Surface	N	Samples/ research field	Aliquot	LOD	Filter/ pore size	Storage T°	WN Range cm^{-1}	Centrifugation	Laser	Spectral Resolution	Cleaning Procedure	Total Procedure Time	Others
1	W. Zhu	2020	Rapid and low-cost quantitative detection of creatinine in human urine with a portable Raman spectrometer		SERS	n.s.	n.s.	Au solution	5	Creatinine	n.s.	1.45 mg L	no	n.s.	500 – 1600	no	785 nm 500 mW<br>1s	n.s.	-	< 2 min	- Cost ¥0.2 each sample<br>- Spiked Urine
2	Y. Ma	2020	Therapeutic prognosis of prostate cancer using surface-enhanced Raman scattering of patient urine and multivariate statistical analysis		SERS	GA-PLS-LDA	n.s.	AgNPs	75	Treatment response of prostate cancer	2 µL	-	no	-80 °C	300 – 1700	10000 rpm for 10min	632.8 nm<br>5 mW<br>20s	n.s.	-	n.s.	Catheter prior to surgery
3	X. Cui	2020	Label-free detection of multiple genitourinary cancers from urine by surface-enhanced Raman spectroscopy		SERS	PCA-LDA	n.s.	silver colloidal solution	158	Detecting different cancers and healthy controls	n.s.	-	Ultrafiltration membrane	-80°C	500 – 1800	14000 rpm for 10min	785 nm<br>5 s	$1 cm^{-1}$	-	n.s.	Fasting urine
4	Y. Si	2020	A novel surface-enhanced Raman scattering-based ratiometric approach for detection of hyaluronidase in urine		SERS	n.s.	n.s.	AuNRs	-	Hyaluronidase activity	40 µL	$1.7 U mL^{-1}$	-	-	1800 – 2200	-	633 nm<br>2mW<br>6s	n.s.	-	n.s.	Spiked Urine
5	A. Gaipov	2020	Development and validation of hybrid Brillouin-Raman spectroscopy for non-contact assessment of mechano-chemical properties of urine proteins as biomarkers of kidney diseases		SERS	n.s.	STATA MP Version 15	AgNPs	100	Assessment of visco-elastic and chemical properties of proteins	n.s.	-	no	-80 °C	n.s.	no	785 nm	n.s	-	-	-
6	M. Steenbeke	2020	Exploring the possibilities of infrared spectroscopy for urine sediment examination and detection of pathogenic bacteria in urinary tractinfections		ATR-FTIR	- PCA<br>- SIMCA	n.s.	-	147	- Bacteria Gram+ and<br>- Lipids<br>- Proteins<br>- Nucleic acids<br>- Carbohydrates<br>- White and red blood cells<br>- Crystals	n.s.	-	no	n.s.	400 – 4000	10 min RT 1439 × g followed by 10 min, RT, 669 × g	-	$4 cm^{-1}$	n.s.	n.s.	Dried urine specimens
7	D. Perez-Guaita	2020	Quantification and Identification of Microproteinuria Using Ultrafiltration and ATR-FTIR Spectroscopy		ATR-FTIR	- SNV<br>- PCA<br>- RMSEP<br>- MCR-ALS	n.s.	-	6	- Haemoglobin<br>- Albumin<br>- Immunoglobulin	0.8 µL	Alb. 6.7 ppm<br>Hb 50 ppm	0.22 µm	n.s.	800 – 4000	14000g for 15 min x3 times	-	$4 cm^{-1}$	n.s.	n.s.	- Dried Urine<br>- Spiked Urine
8	Y. Hong	2020	Optoplasmonic Hybrid Materials for Trace Detection of Methamphetamine in Biological Fluids through SERS		SERS	n.s.	n.s.	SiO_2–AuNPs	-	Methamphetamine	100 µL	$1x10^{-8}$ M	-	n.s.	600 – 1700	no	785 nm<br>10s	n.s.	-	n.s.	-
9	D. Lu	2020	Silver nanocube coupling with a nanoporous silver film for dual-molecule recognition base dultrasensitive SERS detection of dopamine		SERS	n.s	n.s.	AgNF<br>AgNCs	-	Dopamine	<100µL	40 fM	no	n.s.	500 – 1800	10000rpm for 10 min	785 nm	n.s.	-	n.s.	Spiked Urine
10	J.E.L. Villa	2020	Colloidal gold clusters formation and chemometrics for direct SERS determination of bioanalytes in complex media		SERS	- MCR-ALS<br>- PLSR	MATLAB	GNPs	-	- Adenine<br>- Guanine	n.s.	2 µM	n.s.	n.s.	400 – 1800	-	785nm<br>250mW 40 s	$2 cm^{-1}$	-	n.s.	Spike Urine
11	N.E. Markina	2020	Liquid-liquid extraction-assisted SERS-based determination of sulfamethoxazole in spiked human urine		LLE-SERS	n.s.	OriginPro v.8.5	AgNPs	13	Sulfamethoxazole	62 µL	$1.7 mgmL^{-1}$	.	fresh / -80	400-1800 cm^{-1}	2000 g for 3 min	488 nm<br>24 mW<br>10s	n.s.	-	~30 min	Spiked Urine
12	Y.C. Kao	2020	Multiplex Surface-Enhanced Raman Scattering Identification and Quantification of Urine Metabolites in Patient Samples within 30 min		SERS	- PCA<br>- PLSR	n.s.	Ag nanocubes<br>Ag octahedra	40	Miscarriage risk metabolites:<br>- 5β-pregnane-3α, 20α-diol-3α-glucuronide<br>- tetrahydrocortisone	10 µL	n.s	no	n.s.	500 - 1700	no	532 nm 0.2 mW<br>1 s.	n.s.	-	~30 min	-
13	P. Zhang	2019	A Molecular Beacon Based Surface-Enhanced Raman Scattering Nanotag for Noninvasive Diagnosis of Bladder Cancer		SERS	n.s.	n.s.	Sea urchin-like gold nanoparticles	13	- mRNA<br>- Early bladder cancer diagnosis	n.s.	19.4 nM	n.s.	n.s.	n.s.	n.s.	n.s.	n.s.	-	n.s.	-
14	A. Takamura	2019	Phenotype Profiling for Forensic Purposes: Determining Donor Sex Based on Fourier Transform Infrared Spectroscopy of Urine Traces		ATR-FTIR	- PLS-DA<br>- GA	n.s.	-	101	Donor Sex gender	30 µL	-	no	-80 °C	600 – 3700	no	-	$4 cm^{-1}$	70% Ethanol	n.s.	Dried urine
15	Y. Gao	2019	CTAB-triggered Ag aggregates for reproducible SERS analysis of urinary polycyclic aromatic hydrocarbon metabolites		SERS	- Linear double logarithmic equation<br>- RSD	n.s.	AgNPs	1	Polycyclic aromatic hydrocarbon metabolites	n.s.	0.1 µL	n.s.	n.s.	300 – 2000	n.s.	n.s.	n.s.	-	n.s.	Spiked Urine
16	K. Wang	2019	Elucidating fentanyls differentiation from morphines in chemical and biological samples with surface-enhanced Raman spectroscopy		SERS	- PCA<br>- PLSDA	n.s.	AuNPs	3	- Fentanyl<br>- Morphine	200 µL	50 ng/mL	n.s	n.s	400 – 1800	5206rpm for 1min<br>+<br>6000 rpm for 10 min	785nm<br>90 mW<br>10s	n.s.	-	n.s.	Spiked Urine
17	B. Murugesan	2019	Tunable Coffee Ring Formation on Polycarbonate Nanofiber Film for Sensitive SERS Detection of Phenylalanine in Urine		SERS	Linear relationship	n.s.	Polyvinylpyrrolidone (PVP)-stabilized silver colloidal (AgC)	3	Phenylalanine	10 µL	50 µM	no	fresh	500 – 1700	Yes	632.8 nm<br>25 mW<br>1 s	0.06 nm	-	n.s.*	- Spiked Urine<br>- Estimated detection limit 2.35 µM<br>- Dried Urine<br>* at least more than 10 min (time necessary to let the drop dry)

18	H. Yang	2019	Influence of drugs on the prospective diagnostic method for coronary heart disease with urine	SERS	PCA-LDA	n.s.	-	26	Coronary heart disease (PDGF-BB)	n.s.	-	no	4 °C	1200 – 1600	no	785 nm<br>10s	n.s.	-	n.s.	-
19	S. Lee	2019	Diagnosis in a Preclinical Model of Bladder Pain Syndrome Using a Au/ZnO Nanorod-based SERS Substrate	SERS	PCA	XLSTAT 2018	Au/ZnO NRs	-	Bladder pain	5 µL	-	no	n.s.	550 – 1500	no	785nm<br>1mW<br>40s	1 cm^{-1}	-	n.s	-
20	M.T. Alula	2019	Preparation of silver nanoparticles coated ZnO/Fe3O4 composites using chemical reduction method for sensitive detection of uric acid via surface-enhanced Raman spectroscopy	SERS	n.s.	n.s.	AgNPs/ZnO/Fe3O4	-	Uric acid	10 uL	15 µM	no	fresh	400 – 1800	no	532nm<br>7.25mW<br>20s	n.s	-	n.s.*	*> 20min
21	M. Jue	2019	Optimization of ZnO Nanorod-Based Surface Enhanced Raman Scattering Substratesfor Bio-Applications	SERS	- Neural Density<br>- Savitzky–Golay smoothing method	- RAON-Spec<br>- Origin	ZnO	-	Rhodamine B	n.s	-	no	n.s	400 – 1600	no	785-nm<br>1 – 0.1mW<br>50s	1 cm^{-1}	-	n.s	-
22	Z. Huang	2019	Sensitive polydopamine bi-functionalized SERS immunoassay for microalbuminuria detection	SERS	n.s.	- Origin 8<br>- Graph Prism 6.0	polydopamine bi-functionalized	22	Microalbuminuria	2 µL	0.2 mg/L.	no	-20 °C	950 – 1200	no	785 nm<br>4mW<br>1s	n.s.	-	5min	-
23	K. Sivashanmugan	2019	Tetrahydrocannabinol Sensing in Complex Biofluid with Portable Raman Spectrometer Using Diatomaceous SERS Substrates	TLC-SERS	PCA	n.s.	AuNPs	n.s.	Tetrahydrocannabinol	2 µL	10ppm	yes	-	400 – 1800	no	785 nm<br>420 mW<br>10s	n.s.	-	-	- Spiked urine<br>- All samples were analysed within 3 h of collection<br>- Air-dried samples
24	H. Muhamadali	2019	Rapid Detection and Quantification of Novel Psychoactive Substances (NPS) Using Raman Spectroscopy and Surface-Enhanced Raman Scattering	SERS	- PCA<br>- PLSR	MATLAB	Ag colloid	-	- Methcathinones<br>- Aminoindanes<br>- Diphenidines<br>- Synthetic cannabinoids	100 µL	2mM	no	4°C than within 2h<br>−80◦C	100 – 1800	5000g for 10min at 4°C	785 nm<br>60mW<br>20s	n.s.	-	-	- Mid-stream first morning urine samples<br>- Spiked Urine
25	N. Markina	2019	Application of Aluminium Hydroxide for Improvement of Label-Free SERS Detection of Some Cephalosporin Antibiotics in Urine	SERS	RSD	n.s.	AgNPs	5	Cephalosporin	n.s.	20-90 µg/mL	no	4 ◦C	400 – 1800	1700g for 2 min	638nm<br>1s	n.s	-	15min	- Samples were stored at 4 ◦C and used for analysis within one day after collecting<br>- Spiked urine
26	S. Han	2019	Rapid and Sensitive Surface-enhanced Raman Spectroscopy Method for Determination of Ketamine in Urine	SERS	- Correlation coefficient<br>- Linear relationship	n.s.	- AgNPs<br>- NaBr	14	Ketamine	n.s.	7.5 ppm	-	-	175 – 2000	-	785 nm<br>80mW<br>5s	3 cm^{-1}	-	n.s.	-
27	F. Enduo	2019	Dual-Mode Au Nanoprobe Based on Surface Enhancement Raman Scattering and Colorimetry for Sensitive Determination of Telomerase Activity Both in Cell Extracts and in the Urine of Patients	SERS	- Linear relationship<br>- Linear regression equation<br>- Michaelis–Menten equation	n.s.	AuNPs	24	Telomerase Activity	n.s	6.1×10^{-15} IU	n.s	n.s	n.s	n.s	633 nm<br>10 mW	n.s	-	n.s.	-
28	S. Feng	2019	Assessment of treatment efficacy using surface-enhanced Raman spectroscopy analysis of urine in rats with kidney transplantation or kidney disease	SERS	- PCA<br>- LDA<br>- PLS	n.s.	Ag colloids	41	Assessment of treatment efficacy in kidney treatment	5 µL	-	no	− 80 °C	500 – 1800	3000rpm for 10min	785 nm<br>150 mW<br>1s	8 cm^{-1}	-	n.s.	-
29	I. Suzuki	2019	NIR spectroscopic determination of urine components in spot urine: preliminary investigation towards optical point-of-care test	NIR-FT	- Multi-linear regression<br>- Partial least squares regression	n.s.	-	110	Na / Creatinine Ratio	n.s.	-	no	n.s.	4300 – 7150	no	-	n.s.	n.s.	n.s.	-
30	M. Muhammad	2019	Label-free SERS diagnostics of radiation-induced injury via detecting the biomarker Raman signal in the serum and urine bio-samples based on Au-NPs array substrates	SERS	- Linear-Savitzky-Golaywithin.<br>- One-way ANOVA with Tukey's tests	NG-Lab Spec	Au-NPs	5	Radiation-induced injury	n.s.	-	n.s.	n.s.	500 – 1750	no	785 nm	n.s.	-	n.s.	-
31	B. Ankudze	2018	Ultrasensitive and recyclable superstructure of Au SiO2@Ag wire for surface enhanced Raman scattering detection of thiocyanate in urine and human serum	SERS	n.s.	n.s.	Au-SiO2@Ag	-	Thiocyanate	n.s.	0.01 µM	-	-	1800 – 2800	-	785 nm<br>3mW<br>10s	-	-	n.s.	Spiked Urine
32	A. Pallaoro	2018	Screening for canine transitional cell carcinoma (TCC) by SERS-based quantitative urine cytology	SERS	- CLS<br>- Mann–Whitney nonparametric test<br>- receiver operating characteristic curve plotting	- Matlab<br>- PLS-Toolbox<br>OriginPro	AgNP	10	Cancerous transitional cells	7.5 µL		no	−80 °C	250 – 1750	1000g for 3-4 min	633 nm<br>50-90 µW<br>0.5s	n.s	-	-	- Frozen, urine samples were thawed in a 37 °C water bath<br>- After centrifugation urine supernatant was discarded
33	K.M. Koo	2018	Design and Clinical Verification of Surface-Enhanced Raman Spectroscopy Diagnostic Technology for Individual Cancer Risk Prediction	SERS	- Vancouver Raman Algorithm<br>- PCA	SPSS 19.0	AgNPs	120	Prostate cancer risk	61 µL		n.s.	n.s.	400 – 1800	n.s.	785 nm<br>70 mW<br>1s	n.s.	-	90 min	-

34	Yuan Xiang	2018	Surface enhanced Raman detection of the colon cancer biomarker cytidine by using magnetized nanoparticles of the type Fe3O4/Au/Ag			SERS	Linear relationship	n.s.	Fe3O4/Au/Ag	n.s.	Cytidine	100 µL	1 nM	n.s.	-	400 – 2000	n.s.	633 nm<br>5 mW<br>8 s	n.s.	-	n.s.	Spiked Urine
35	X. Lin	2018	A novel urine analysis technique combining affinity chromatography with Au nanoparticle based surface enhanced Raman spectroscopy for potential applications in non-invasive cancer screening			SERS	- PCA-LDA<br>- ROC curve	WIRE 3.4	AuNPs	151	Detection of modified nucleoside in human urine	2 µL	-	no	−80 °C	600 – 1700	12000rpm for 10 min	785 nm<br>2.3 mW<br>10s	2 cm^{-1}	-	n.s.	12 hours of overnight fasting before colection
36	M. Paraskevaidi	2018	Potential of mid-infrared spectroscopy as a non invasive diagnostic test in urine for endometrial or ovarian cancer			ATR-FTIR	- PLS-DA<br>- PCA-SVM<br>- GA-LDA	PLS Toolbox 7.9.3	-	30	- Endometrial cancer<br>- Ovarian cancer	50 µl	-	no	−80 °C	800 – 1800	no	-	8 cm^{-1}	Distilled water	n.s.	-6 hours fasting prior surgery<br>-Samples are left to air-dry for approximately 45 min
37	I. Suzuki	2018	Reagentless Estimation of Urea and Creatinine Concentrations Using Near-Infrared Spectroscopy for Spot Urine Test of Urea-to-Creatinine Ratio			NIR-FT	- leave-one-out cross-validation<br>- SEP<br>- Correlation coefficient (γ)	R programming version 3.3.1	-	110	- Urea<br>- Creatinine	n.s.	-	no	n.s.	4300 – 7150	no	-	1.667 nm	-	30 s	- Measures at room temperature of 25°C<br>- Spiked Urine
38	L.P. Moreira	2018	Detecting urine metabolites related to training performance in swimming athletes by means of Raman spectroscopy and principal component analysis			RS	- PCA<br>- ANOVA	- Matlab 7.4<br>- RamanSoft 1.04	-	23	- Urea<br>- Creatinine<br>- Ketone bodies<br>- Nitrogenous compounds<br>- Phosphate	80 µL	-	no	-20°C	400 – 1800	no	830nm<br>350mW<br>20s	4 cm^{-1}	-	n.s.	Frozen urine samples were passively warmed up to room temperature (25°C)
39	Q. Zhu	2018	Antipsychotic drug poisoning monitoring of clozapine in urine by using coffee ring effect-based surface-enhanced Raman spectroscopy			CRE-SERS	- Savitzky-Golay polynomial fitting<br>- Density functional theory	- Matlab<br>- Origin 7.5	Ag colloids	n.s	Clozapine	1 µL	0.5µg mL^{-1}	no	-20°C	350 – 2300	10000rpm for 10min	785 nm<br>200mW<br>5s	5 cm^{-1}	-	-	-
40	M.E. Markina	2018	Sample pretreatment and SERS-based detection of ceftriaxone in urine			SERS	Calibration plots	n.s.	AgNPs	-	Ceftriaxone	10 µL	0.4 µg/mL	-	-	300 – 2000	no	473 nm<br>5mW<br>10 s	3 cm^{-1}	-	~ 10 min	Spiked Urine
41	Z. Mukanova	2018	Detection of Paracetamol in Water and Urea in Artificial Urin e with Gold Nanoparticle@Al Foil Cost-efficient SERS Substrate			SERS	Calibration curve	n.s.	AuNPs		Urea	25 µL	0.026 M	-	-	800 – 1400	-	633 nm<br>1.5 - 3 mW<br>5s	n.s.	-	-	Spiked Urine
42	H. Yang	2018	Non invasive and prospective diagnosis of coronary heart disease with urine using surface-enhanced Raman spectroscopy			SERS	- PCA-LDA<br>- Leave-one-out cross validation<br>- Savisky-Golay smoothing	n.s.	-	107	- Diagnosis of coronary heart disease<br>- PDGF-BB	0.5 ml	0.01 ppm	no	4 °C	1140 – 1620	no	785 nm<br>10s	0.7 cm^{-1}	-	-	-The urine samples were obtained before performing PCI<br>-Urine was collected in the morning at 8 o'clock
43	S. Weng	2018	Dynamic surface-enhanced Raman spectroscopy and Chemometric methods for fast detection and intelligent identification of methamphetamine and 3, 4-Methylenedioxy methamphetamine in human urine			D-SERS	- PCA<br>- Random forest	n.s.	mPEG-SH coated gold nanorods (GNRs)	550	- Methamphetamine<br>- 3, 4-Methylenedioxy methamphetamine	2 µL	0.4 ppm	n.s.	n.s.	600 – 1800	n.s.	785 nm<br>5s	8 cm^{-1}	-	2 mins	-Spiked urine<br>-During the state from wet to dry, 20 spectra were obtained
44	Yu B.	2018	Sensitive and simple determination of zwitterionic morphine in human urine based on liquid-liquid micro-extraction coupled with surface-enhanced Raman spectroscopy			SERS	RSD	n.s.	Au NRs	50	Morphine	n.s.	1 ppm	-	4 °C then −80°C°C within 2h	400 – 1800 cm^{-1}	5 min at 6000 rpm	785 nm<br>5s	n.s.	-	6min	Spiked Urine
45	C. Zhang	2018	Detection and Quantification of Bucinnazine Hydrochloride Injection Based on SERS Technology			SERS	Boxcar averaging	Bwram 1.01.20	AgNPs	-	Bucinnazine Hydrochlorid	60 µL	0.5 µg/mL	-	-	175 – 2000	-	785 nm<br>75mW<br>5s	3 cm^{-1}	-	n.s.	Spiked Urine
46	S. Bindesri	2018	Development of an electrochemical surface-enhanced Raman spectroscopy (EC-SERS) fabric-based plasmonic sensor for point-of-care diagnostics			SERS	n.s.	Origin 9.0	AgNPs	-	Levofloxacin	n.s.	0.1 M	-	-	200 – 1800	-	785 nm<br>46.5 mW<br>30s	5 cm^{-1}	-	n.s.	Spiked Urine
47	D. Li	2018	Griess reaction-based paper strip for colorimetric/fluorescent/SERS triple sensing of nitrite			SERS	n.s.	n.s.	GNRs-Azo-GNPs	-	Nitrite	10 µL	0.8 nM	n.s.	n.s.	200 – 1000	n.s.	785 nm<br>10mW<br>10s	5 cm^{-1}	-	n.s.	Spiked Urine
48	Y. Lu	2018	Diazotization-Coupling Reaction-Based Determination of Tyrosine in Urine Using AgNanocubes by Surface-Enhanced Raman Spectroscopy			SERS	Recovery test	n.s.	AgNCs	-	Tyrosine	n.s.	10^{-8} M	no	-	1000 – 1800	yes	78 5nm<br>90 µW<br>0-1200s	n.s.	-	n.s.	-Morning urine<br>-Spiked Urine
49	Y. Lu	2018	Fabrication and Characterization of a Highly Sensitive Surface-Enhanced Raman Scattering Nanosensor for Detecting Glucose in Urine			SERS	n.s.	n.s.	Au@Ag NPs	-	Glucose	n.s.	0.1mM	n.s.	n.s.	800 – 1800	n.s.	785 nm<br>0.34 mW<br>10s	n.s.	-	n.s.	-Samples dry at room temperature<br>-Spiked Urine
50	N. Yamamoto	2018	Ultrasonic standing wave preparation of a liquid cell for glucose measurements in urine by midinfrared spectroscopy and potential application to smart toilets			NIR-FT	- Standard deviation correlation<br>- Correlation coefficient	n.s.	-	1	Glucose	n.s.	50 mg/dL	no	no	714 – 1250	no	-	n.s.	-	n.s.	Spiked Urine
51	C. Zheng	2018	Silver nanoparticles/activated carbon composite as a facile SERS substrate for highly sensitive detection of endogenous formaldehyde in human urine by catalytic reaction			SERS	Liner correlation	n.s.	Ag NPs /AC composite	5	Formaldehyde	10 µL	0.07 ppm	n.s.	−70 °C	400 – 4000	n.s.	633 nm<br>6mW<br>5s	2 cm^{-1}	-	n.s.	Morning urine

52	J.E.L. Villa	2018	Surface-enhanced Raman spectroscopy and MCR-ALS for the selective sensing of urinary adenosine on filter paper			SERS	MCR-ALS	n.s.	GNPs	3	Adenosine	200 µL	3.8–4.9 µmol L^{-1}	0.45 µm	−4 °C	400 – 1800	no	785 nm 125 mW<br>20 s	0.05 mm	-	6 min	-
53	A. Stefancu	2018	SERS-based quantification of albuminuria in the normal-to-mildly increased range			SERS	- PCA-LDA<br>- PLSR	Unscrambler X (CamoAnalytics)	Iodide modified AgNPs (IMNPs)	27	Albumin	5 µL	3 µgm L^{-1}	no	−20 °C	0 – 1700	5800g for 15 min	633 nm<br>30 mW<br>40s	n.s.	-	n.s.	-
54	V. Turzhitsky	2018	Pico analysis of Drugs in Biofluids with Quantitative Label-Free Surface-Enhanced Raman Spectroscopy			SERS	Semiempirical Quantitative Algorithm	n.s.	Gold Nanoparticle	n.s.	Opiods	10 µL	100 ng mL^{-1}	n.s.	n.s.	100 – 3100	n.s.	785 nm<br>200 mW	4-5 cm^{-1}	-	>2min*	- Spiked Urine<br>- For concentrations <1000 ng mL−1 the extraction procedure requires additional 20min
55	K. Mao	2018	A novel biosensor based on AuAg core-shell nanoparticles for sensitive detection of methylamphetamine with surface enhanced Raman scattering			SERS	Logarithm linear correlation	n.s.	Au@Ag	n.s.	Methylamphetamine	100 µL	0.16 ppb	0.22 µm	n.s.	800 – 1800	n.s.	532 nm<br>10s	n.s.	Aqua regia (HCl/HNO3) and rinsed with ultrapure water throughout the experiment	n.s.	Spiked Urine
56	S. Feng	2017	A non invasive cancer detection strategy based on gold nanoparticle surface-enhanced raman spectroscopy of urinary modified nucleosides isolated by affinity chromatography			SERS	PLS-DA	n.s.	AuNPs	169	Modified nucleosides for nasopharyngeal cancer and esophageal cancer	n.s.	-	no	−4 °C	600 – 1700	no	785 nm<br>5mW<br>10s	2 cm^{-1}	-	n.s.	Samples were processed within 48 h after collection.
57	J. Wang	2017	A nanoplasmonic label-free surface-enhanced Raman scattering strategy for non-invasive cancer genetic subtyping in patient samples			SERS	- PCA-LDA<br>- ROC analysis	SPSS 19.0	AgNPs	43	- Simple nucleic acid (DNA/RNA)<br>- Prostate cancer	61 µL	-	Yes (ZRC GF™)	n.s.	400 – 1800	n.s	785 nm<br>70mW<br>1 s	n.s	n.s.	~2 h	-
58	C. Westley	2017	Absolute Quantification of Uric Acid in Human Urine Using Surface Enhanced Raman Scattering with the Standard Addition Method			SERS	Standard addition	n.s.	Silver colloid	58	- Uric Acid<br>- Preeclampsia	125 µL	-	no	n.s.	0 – 3000	no	633 nm<br>3 mW<br>20 s	n.s.	-	140 s	Clinical prepreeclamptic patients provided urine samples collected between 11 and 14 weeks gestation
59	T. Mostowtt	2017	Surface enhanced Raman spectroscopy (SERS) as a method for the toxicological analysis of synthetic cannabinoids			SERS	n.s.	n.s.	AgNPs	-	Synthetic cannabinoids	10 µL	18 ng/ mL	-	-	300 – 1800	-	785 nm l00 mW	4 cm^{-1}	-	n.s.	Spiked Urine
60	L.P. Moreira	2017	Raman spectroscopy applied to identify metabolites in urine of physically active subjects			RS	Lorentzian peak fitting function	Microcal Origin 6.0	-	14	Metabolites	80 µL	-	no	-20°C	400 – 1800	no	830nm<br>350mW<br>20s	2 cm^{-1}	-	n.s.	-
61	Premasiri W.R.	2017	Rapid urinary tract infection diagnostics by surface-enhanced Raman spectroscopy (SERS): identification and antibiotic susceptibilities			SERS	PLS-DA	Matlab	Au nanoparticle-covered SiO2 substrate	12	Bacterial identification	50 µL	-	5-µm nylon mesh sheets (Cole-Palmer)	n.s.	400 – 1800	10000rpm for 20min	785 nm<br>0.45 mW<br>10s	n.s.	-	~40 min	Spiked Urine
62	A. Kamińska	2017	Gold-capped silicon for ultrasensitive SERS-biosensing: Towards human biofluids analysis			SERS	- Savisky-Golay smoothing method<br>- Correlation Coefficent	n.s.	Au/Si surface	-	- Cholesterol<br>- Uric acid<br>- Albumin<br>- Urea<br>- Phenylalanine tryptophan<br>- D-Galactosamine hydroxyproline	15 µL	n.s.	n.s.	n.s.	600 – 1700	n.s.	785nm<br>5 mW<br>4-10s	5-6 cm^{-1}	-	n.s.	-
63	M.K. Nguyen	2017	A Plasmonic Coupling Substrate Based on Sandwich Structure of Ultrathin Silica-Coated Silver Nanocubes and Flower-Like Alumina-Coated Etched Aluminum for Sensitive Detection of Biomarkers in Urine			SERS	Linear correlation	n.s.	Ag@SiO2 NCs	-	- Creatinine<br>- Flavin adenine dinucleotide	20 µL	5 ×10^{-5} M	-	4 °C	500 – 1800	-	532 nm<br>1s<br>1.5 mW	1 cm^{-1}	-	n.s.	-
64	I. Gregório	2017	Analysis of human bodily fluids on superabsorbent pads by ATR-FTIR			ATR-FTR	n.s.	OriginPro 8	-	3	- Semen<br>- Vaginal fluid<br>- Urine	0.05 mL	-	no	n.s	800 – 2000	no	-	4 cm^{-1}	- isopropanol	n.s.	All stains were left to dry overnight in a biological safety cabinet (BSC) prior to analysis
65	M.C. Yu	2017	Label Free Detection of Sensitive Mid-Infrared Biomarkers of Glomerulonephritis in Urine Using Fourier Transform Infrared Spectroscopy			ATR-FTR	- PCA<br>- one-way Analysis of Variance<br>- Fisher's Least Difference Significance	Origin Pro 9.1	-	35	Glomerulonephritis	5 µl	-	no	none (Analysis was done soon after collection)	800 –4000	1800 rpm<br>4 °C<br>for 10 min	-	4 cm^{-1}	Distilled water and ethanol	n.s.	Samples are dried on to the ATR prism surface with nitrogen gas
66	Q. Chena	2017	Highly sensitive detection of glucose: A quantitative approach employing nanorods assembled plasmonic substrate			SERS	n.s.	n.s.	Au@Ag NRs	n.s.	Glucose	n.s.	10^{-8} M	n.s.	n.s.	300 – 2500	5000rpm for 5min	633 nm<br>10s	n.s.	-	n.s.	Spiked urine
67	J. Chi	2017	Surface-enhanced Raman scattering analysis of urine from deceased donors as a prognostic tool for kidney transplant outcome			SERS	- PCA-LDA<br>- Logistic regression<br>- Chi-square test	- Matlab<br>- SAS Software	AgNPs	30	Kidney injury	1.5 uL		no	n.s	500 – 1800	no	632.8 nm<br>0.5 mW<br>20s	n.s.	-	n.s.*	* ca. <1min
68	J. Menga	2017	Designing of ordered two-dimensional gold nanoparticles film for cocaine detection in human urine using surface-enhanced Raman spectroscopy			SERS	n.s	n.s.	GNPs	n.s.	Cocaine	10 µL	500 ppb	n.s.	n.s.	600 – 1800	n.s.	785nm<br>270mW<br>10 s	n.s.	-	3 min	Spiked Urine
69	S. Jaychandran	2016	Raman Spectroscopic Analysis of Blood, Urine, Saliva and Tissue of Oral Potentially Malignant Disorders and Malignancy-A Diagnostic Study			RS	PCA-LDA	SPSS/PC +19	-	158	Healthy, pre- and malignancy oral cancer patients	n.s.	-	n.s.	n.s.	400 – 1800	n.s.	785 nm<br>100mW	n.s.	-	n.s.	-

70	Gok S.	2016	Bladder cancer diagnosis from bladder wash by Fourier transform infrared spectroscopy as a novel test for tumor recurrence	ATR-FTIR	- PCA<br>- Hierarchical cluster analysis<br>- Multivariate Analysis software<br>- Ward's algorithm<br>- Mann-Whitney U test	- Spectrum 100<br>- Unscrambler ® X Version 10.3 (CAMO)<br>- OPUS 5.5	-	136	Bladder cancer	n.s.	-	no	–80 °C	4000 – 650	10000rpm for 30min	-	4 cm^{-1}		n.s.	- Liquid bladder wash samples taken from –80 °C were thawed, directly put on the ATR crystal and scanned<br>- Di/ZnSe crystal plate
71	Cheng J.	2016	Highly Sensitive Detection of Clenbuterol in Animal Urine Using Immunomagnetic Bead Treatment and Surface-Enhanced Raman Spectroscopy	SERS	- Savitzky-Golay second derivative transformation<br>- Polynomial subtractions smoothening	RamanAnalyzer	GO/AuNPs	40	Clenbuterol	2 mL	0.5 ng·mL^{-1}	no	4 °C	500 – 2500	no	785nm<br>200 mW<br>10 s	4 cm^{-1}	-	n.s.	-
72	R. Colleen	2016	Sheath flow SERS for chemical profiling in urine	SERS	Pearson correlation coefficient	Matlab	Ag surface	-	Benzoylecgonine	1.5 µL	30 ng mL^{-1}	n.s.	n.s.	800 – 1800	n.s.	660 nm<br>3-4mW<br>200-250ms	n.s.	nanopure water and borate buffer	n.s.	Spiked urine
73	K.V. Oliver	2016	Effects of the Hydration State on the Mid-Infrared Spectra of Urea and Creatinine in Relation to Urine Analyses	FTIR	Curve fitting analysis with Gaussian or pseudo-Voigt functions	- Origin 8.6<br>- Gaussian 09	-	5	- Urea<br>- Creatinine	3 µL	-	no	No, fresh	800 – 4000	no	no	4 cm^{-1}	- n.s.	<7min	Data were recorded at room temperature
74	Y.S. Selbe	2016	Surface-enhanced Raman probe for rapid nano extraction and detection of erythropoietin in urine	SERS	Relative standard deviation of calibration curve	n.s.	Au–Fe3O4 (MGNPs)<br>GNRs	-	Erythropoietin	10 µL	0.1 pg mL^{-1}	n.s.	n.s.	200 – 2000	n.s.	785-nm<br>150mW<br>60s	n.s.	-	n.s.*	- Spiked Urine<br>-Study, the cost < US$30<br>-* total analysis time lasts around 2 days including particle generation and antibody immobilization.
75	P. She	2016	A competitive immunoassay for ultrasensitive detection of Hg(2+) in water, human serum and urine samples using immunochromatographic test based on surface-enhanced Raman scattering	SERS	n.s.	n.s.	GNPs	-	Mercury	100 mL	0.45 pg mL^{-1}	no	n.s.	0 – 2000	no	785nm<br>40 mW<br>5s	n.s.	-	-	Spiked Urine
76	J.E.L. Villa	2016	A portable SERS method for the determination of uric acid using a paper-based substrate and multivariate curve resolution	SERS	MCR-ALS	n.s.	GNPs	-	Uric acid	n.s.	0.11 $mmolL^{-1}$	-	-	400 – 1800	-	785 nm<br>75W<br>1.5-2s	12-14 cm^{-1}	-	15- 80min	- Spiked Urine<br>- Substrates were dipped for 15 min and dried prior to analysis
77	Y. Ma	2016	Surface-Enhanced Raman Spectroscopy on Liquid Interfacial Nanoparticle Arrays for Multiplex Detecting Drugs in Urine	SERS	Linear relationship	n.s.	GNPs	-	- Methamphetamine<br>- 3,4-Methylenedioxy methamphetamine (MDMA)	n.s.	0.5 ppm	no	n.s.	600 – 1300	no	633 nm<br>0.5 mW<br>5s	n.s.	-	n.s	- Spiked Urine<br>- Human urine was rapidly separated and purified in 3 min
78	K.V. Oliver	2016	Infrared vibrational spectroscopy: a rapid and novel diagnostic and monitoring tool for cystinuria	ATR-FTIR	- Pearson correlation coefficient<br>- Bland–Altman plot	OPUS 6.5	-	27	Cystine	5 µl	3 mM	no	-20°C	750 – 4000	no	-	4 cm^{-1}	n.s.	n.s.	-Midstream urine collection<br>- Spectra recorded at room temperature<br>- Spectra of 'as-collected' undried urine
79	Cassiano	2016	Quantifying creatinine and urea in human urine through Raman spectroscopy aiming at diagnosis of kidney disease	RS	- PLS<br>- Correlation coefficient (r)<br>- Root mean square error of cross-validation	- RamanSoft version 1.7<br>- Matlab 6.0<br>- Chemoface toolbox	-	54	- Creatinine<br>- Urea	100 µL	-	no	-20 °C<br>(-78 °C for transport)	400 – 1800	no	830nm<br>350mW<br>30s	2 cm^{-1}	-	n.s.	Midstream urine
80	K. Kinoshita	2016	Detection of urinary estrogen conjugates and creatinine using near infrared spectroscopy in Bornean orangutans (Pongo Pygmaeus)	NIR	- PLS<br>- Savitzky–Golay second derivative	- OPUS<br>- CAMO	-	173	- Estrogen<br>- Creatinine	100 µL		no	-20°C	4000 – 12500	yes	-	8 cm^{-1}	-	n.s.	Spectra measured at room temperature (25 °C)
81	X. Gu	2016	Sensing Glucose in Urine and Serum and Hydrogen Peroxide in Living Cells by Use of a Novel Boronate Nanoprobe Based on Surface-Enhanced Raman Spectroscopy	SERS	n.s.	Winspec 32	3-MPBA - AuNPs	-	Glucose	1-10 µL	70 nM	-	-	350 – 1700	-	785 nm<br>1.3mW<br>5s	n.s.	-	<20min	Spiked Urine
82	A. Subaihi	2016	Rapid, Accurate, and Quantitative Detection of Propranolol in Multiple Human Biofluids via Surface-Enhanced Raman Scattering	SERS	- PCA<br>- PC-DFA<br>- PLSR	n.s.	AgNPs	-	Propranolol	200 µL	168.6 ng/mL<br>(0.57 µM)	no	–80 C°	400 – 2000	5000g at 4 °C for 10min	785nm<br>60mW<br>20s	n.s.	-	n.s.	- Midstream first morning<br>- Spiked Urine<br>- urine briefly kept at 4 C°, frozen within 2h
83	I.J. Hidi	2016	Lab-on-a-Chip-Surface Enhanced Raman Scattering Combined with the Standard Addition Method: Toward the Quantification of Nitroxoline in Spiked Human Urine Samples	SERS	- Selective nonlinear iterative peak clipping<br>- MCR-ALS	Internal house written algoritmus (GnuR)	AgNPs	10	Nitroxoline	n.s.	3.43 µM<br>(0.57 mg/L)	0.22 µm	–21 °C	200 – 1650	no	532nm<br>13 mW<br>1s	1 cm^{-1}	-	n.s.	-Spiked Urine<br>-Urine were kept in the freezer for 8 months before the measurements were performed.
84	K.M. Koo	2016	Rapid and Sensitive Fusion Gene Detection in Prostate Cancer Urinary Specimens by Label-Free Surface-Enhanced Raman Scattering	SERS	Calibration Curve	n.s.	AuNPs	3	TMPRSS2-ERG	30-50 ml*	10^3 copies	no	n.s.	600 – 1800	700g for 10min	785 nm<br>70 mW<br>1s	n.s.	-	75min**	-* for RNA Extraction procedure, 11µL volume for SERS<br>- **Extraction +SERS

85	K.M. Koo	2016	Toward Precision Medicine: A Cancer Molecular Subtyping Nano-Strategy for RNA Biomarkers in Tumor and Urine	SERS	Normalized Raman intensities for quantitative comparisons	n.s.	AuNPs	n.s.	RNA targets for Prostata Ca	100 µL	≈100 copies	yes	n.s.	400 – 1500	no	785 nm<br>70w<br>2s	n.s.	-	80 min*	-*overall Time with sample preparation and RNA extration
86	B. Elumalai	2015	Raman spectroscopic characterization of urine of normal and oral cancer subjects	RS	PCA-LDA	Labspec	-	167	Oral cancer	n.s.	-	no	4 °C	500 – 1800	no	785nm<br>13mW<br>90 s	n.s.	-	n.s.	-First voided morning urine<br>-Samples thawed to room temperature<br>-The samples were examined within 48h
87	G. Del Mistro	2015	Surface-enhanced Raman spectroscopy of urine for prostate cancer detection: a preliminary study	SERS	PCA-LDA	- hyperSpec package<br>- R	AuNPs	18	Prostata cancer	5 µL	-	3 kDa	−80 °C	400 – 1800	4000g at 4 °C for 15min	85nm<br>10 s<br>120 mW	n.s.	-	n.s.	-Morning urine samples<br>-Samples frozen within 5h<br>-Urine samples thawed at 37 °C in water bath
88	J. Chi	2015	Use of surface-enhanced Raman scattering as a prognostic indicator of acute kidney transplant rejection	SERS	n.s.	OriginPro 8.5	AgNPs	58	Acute kidney transplant rejection	n.s.	-	n.s.	−80°C	300 – 2000	n.s.	632.8 nm<br>5mw<br>20s	n.s.	-	n.s.	-
89	U.C. Schröder	2015	Rapid, culture-independent, optical diagnostics of centrifugally captured bacteria from urine samples	RS	n.s.	- R<br>- hyperSpec and “cbmodels Package<br>- MATLAB<br>- PLS toolbox	-	-	Bacteria	4 µL	-	5nm	n.s.	300 – 1800	11500g for 5min	532nm<br>35mw<br>5s	n.s.	-	100 min*	* Total duration of all of the above process steps, from the initial arrival of the patient sample into the lab, to the identification of the pathogen using Raman spectroscopy
90	M. Li	2015	Reagent- and separation-free measurements of urine creatinine concentration using stamping surface enhanced Raman scattering (S-SERS)	S-SERS	n.s.	- Winspec<br>- MATLAB	Nanoporous gold disk	-	Creatinine	1 µL	0.68 mg/dl	no	n.s.	550 – 1650	no	785nm<br>30 mW<br>20sec	n.s.	-	1h*	*The entire assay from warming up reagents to room temperature to calculating creatinine concentration
91	O. Alharbi	2015	Detection and quantification of the opioid tramadol in urine using surface enhanced Raman scattering	SERS	n.s	- DeltaNu<br>- NuSpec	Silver hydroxylamine	-	Tramadol	n.s.	2.5×10^{-6}<br>(657.5 ng mL^{-1})	-	-	200 -2000<br>(potentially 0 -3500)	-	633 nm<br>30 mW	8 cm^{-1}	-	n.s.	-
92	R. Dong	2015	Detection and Direct Readout of Drugs in Human Urine Using Dynamic Surface-Enhanced Raman Spectroscopy and Support Vector Machines	SERS	- SVM<br>- Radial basis function<br>- PCA	MATLAB	GNRs	53	- MAMP<br>- MDMA	2 µL	0.1 ppm	no	n.s.	600 – 1800	no	785nm<br>3.5 mW<br>3s<br>/<br>785nm<br>120mW<br>5s	n.s.	-	2min	-
93	Z. Han	2015	Portable Kit for Identification and Detection of Drugs in Human Urine Using Surface-Enhanced Raman Spectroscopy	SERS	RSD	n.s.	GNRs	30	- MAMP<br>- MDMA	2 µL	0.1 ppm	no	n.s.	200 – 2000	no	785 nm<br>100 mW<br>15 s	n.s	-	ca. 5min	
94	Z. Han	2015	Three-Dimensional Surface-Enhanced Raman Scattering Hotspots in Spherical Colloidal Superstructure for Identification and Detection of Drugs in Human Urine	SERS	- SVM<br>- PCA	n.s.	AgNPs	4	Methamphetamine	1 µL	10 ppb	n.s.	n.s.	600 – 1250	10000 rpm for 2min	n.s.	n.s.	-	n.s.*	-*> 5min
95	S. Clauson	2015	Detection of Pesticides and Metabolites Using Surface-Enhanced Raman Spectroscopy (SERS): Acephate	SERS	n.s.	- Grams AI<br>- Grams IQ<br>- Origin 8.5	Ag Active Surface	-	Acephate	0.4 or 5 µL	1ppb	n.s.	n.s.	200 – 3400	n.s.	785-350nm<br>150mW<br>/<br>785nm<br>120 mW	1 cm^{-1}	-	<1min*	-Spiked Urine<br>-*only spectra acquisition
96	E.L. Doctor	2015	The application of supported liquid extraction in the analysis of benzodiazepines using surface enhanced Raman spectroscopy	SERS	- Linear dynamic ranges<br>- Calibration curves	n.s.	Au colloids	-	Benzodiazepines	n.s.	32ng/mL	-	-	400 – 1800	-	785nm<br>10 s<br>100 mW	n.s.	-	< 20min	Spiked Urine
97	R.A. Karaballi	2015	Development of an electrochemical surface-enhanced Raman spectroscopy (EC-SERS)aptasensor for direct detection of DNA hybridization	SERS	n.s.	Origin 8.1	AgNPs	-	DNA hybridization	10 µL	280 mg mL^{-1}	-	-	200 – 2000	-	780 /785 nm<br>22.3-55.9 mW<br>30-60s	5 cm^{-1}<br>/<br>3 cm^{-1}	-	n.s.	-
98	T. Yang	2014	Facile and Label-Free Detection of Lung Cancer Biomarker in Urine by Magnetically Assisted Surface-Enhanced Raman Scattering	SERS	- Student t test<br>- Student’s t-values	n.s.	Fe3O4/Au/Ag NPs	3	Adenosine	5 µL	0.1 µM	no	n.s,	500 – 1800	10000rpm for 10min	632.8nm<br>5mW<br>/<br>785nm<br>300mW	n.s.	-	n.s.	-Spiked Urine<br>-Samples equilibrate for 30 min at room temperature
99	I. Syed	2014	Quantitation of urea in urine by Fourier transforms infrared spectroscopy	FTIR	Linear relationship	n.s.	-	-	Urea	n.s.	1.25 µg/mL	no	n.s.	400 – 4000	no	-	4 cm^{-1}	n.s.	32s*	- Spiked Urine<br>- *scanning Time
100	K.V. Kong	2014	A Rapid and Label-free SERS Detection Method for Biomarkers in Clinical Biofluids	SERS	PCA	Matlab 7.0	BMFON chips	9	- Bladder cancer<br>- Alpha-1 Antitrypsi	10 µL	-	n.s.	−20 °C	1000 – 2400	n.s.	633nm<br>10 s<br>6.2 mW	3 cm^{-1}	-	n.s.	Samples were incubated for 1 h with the anti-A1AT
101	K.V. Kong	2014	Sensitive SERS-pH sensing in biological media using metal carbonyl functionalized planar substrates	SERS	n.s.	WiRE3.0 software	AuNPs	-	pH	20 µL	-	n.s.	-20 °C	100 – 2200	n.s.	633nm<br>0.6 mW<br>10s	n.s.	-	n.s.	Spiked Urine

102	M.T. Alua	2014	Photochemical decoration of magnetic composites with silvernanostructures for determination of creatinine in urine by surface-enhanced Raman spectroscopy			SERS	Linear correlation	n.s.	Ag@ZnO/Fe3O4	-	Creatinine	n.s.	25 nM	n.s.	n.s.	400 – 1800	n.s.	632.8-nm<br>35mW<br>5s	n.s.	-	n.s.	-
103	M. Li	2014	Ultrasensitive and Quantitative Detection of a New β-Agonist Phenylethanolamine A by a Novel Immunochromatographic Assay Based on Surface-Enhanced Raman Scattering (SERS)			SERS	Recovery Test	n.s.	Au@Ag	-	β-Agonist Phenylethanolamine	n.s.	0.32 pg mL^{-1}	n.s.	no	500 – 2000	n.s.	785nm<br>20 mW<br>10 s	n.s.	-	n.s.*	*>15min
104	L. Zhao	2014	Quantitative Detection of Uric Acid by Electrochemical-SurfaceEnhanced Raman Spectroscopy Using a Multilayered Au/AgSubstrate			SERS	- Linear regression analysis<br>- Relative Error	n.s.	Au/Ag substrate	-	Uric Acid	2 µL	0.1 M	-	-	200 – 1800	-	532 nm.<br>1mW<br>30 s	3 cm^{-1}	-	n.s.	-
105	K.V. Kong	2014	Sensitive SERS glucose sensing in biological media using alkyne functionalized boronic acid on planar substrates			SERS	n.s.	WiRE3.0	Au/Ag polystyrene	-	Glucose	n.s.	100 µM	n.s.	-20 °C	500 – 2300	n.s.	785nm<br>300 mW<br>10s	n.s.	-	n.s.	-
106	G. Cao	2014	Quantification of an exogenous cancer biomarker in urinalysis by Raman Spectroscopy			SERS	n.s.	n.s.	β-CD functionalized Klarite		Acetyl amantadine (AcAm)	n.s.	1 ng mL^{-1}	-	-	0 – 2000	-	785 nm<br>23 mW<br>30s	n.s.	-	n.s.	-
107	M.B. Mamián-López	2013	Quantification of moxifloxacin in urine using surface-enhanced Raman spectroscopy (SERS) and multivariate curve resolution on a nanostructured gold surface			SERS	- MCR-ALS<br>- Savitzky-Golay second derivative	n.s.	Nanostructured gold substrate	3	Moxifloxacin	0.50 µL	0.26 µgmL^{-1}	0.22-µm	no	200 – 2200	no	785 nm<br>250mW<br>8 s	4 cm^{-1}	-	n.s.	Early morning urine
108	A.R. Khaskheli	2013	Estimation of ibuprofen in urine and tablet formulations by transmission Fourier Transform Infrared spectroscopy by partial least square			FTIR	- PLS<br>- RMSEC<br>- RMSECV<br>- RMSEP	OMNIC 7.3	-	-	Ibuprofen	n.s.	0.77 lgml^{-1}	n.s.	n.s.	400 – 4000	n.s.	-	4 cm^{-1}	n.s.	n.s.	-Urine samples were collected from healthy volunteers after 2 h the doze of an ibuprofen tablet<br>-Spiked urine
109	B.L. Goodall	2013	Electrochemical-surface enhanced Raman spectroscopy(E-SERS) of uric acid: a potential rapid diagnostic method for early preeclampsia detection			n.s.	n.s.	n.s.	Ag NPs	-	Uric acid	n.s.	<1mM	-	-	200 – 2000	-	785nm<br>46.5 mW<br>30s	n.s.	-	n.s.	-
110	R. Huang	2013	Detection of tobacco-related biomarkers in urine samples by surface-enhanced Raman spectroscopy coupled with thin-layer chromatography			SERS	Calibration curve	n.s.	AgNPs	-	Trans-3′hydroxycotinine (3HC)	5 µL	10 nM	n.s.	n.s.	200 – 2000	2500rpm for 15min	514 - 647 - 785nm<br>10 mW<br>10s	n.s.	-	n.s.	Spiked Urin
111	E.L. Doctor	2013	Comparison of aggregating agents for the surface-enhanced Raman analysis of benzodiazepines			SERS	Calibration curve	n.s.	Gold colloids	-	Benzodiazepines	10 µL	2.5 ng mL^{-1}	n.s	n.s	400 – 1800	n.s.	785nm<br>100 mW<br>10s	n.s.	-	n.s.	Spiked Urin
112	U.C. Schröder	2013	Combined Dielectrophoresis–Raman Setup for the Classification of Pathogens Recovered from the Urinary Tract			DEP-RS	- LDA<br>- PCA	- R<br>- HyperSpec Package	-	3	Bacteria	200 µL		2.7 µm	n.s.	600 – 1800	11500g for 5min	532nm<br>15mW<br>10s	n.s.	-	35min	-
113	S. Kloss	2013	Culture Independent Raman Spectroscopic Identification of Urinary Tract Infection Pathogens: A Proof of Principle Study			Raman-microscopy	SVM	R	-	10	- Enterococcus faecalis<br>- Enterococcus faecium<br>- Staphylococcus epidermidis<br>- Staphylococcus haemolyticus<br>- Staphylococcus hominis<br>- Staphylococcus saprophyticus<br>- Staphylococcus aureus<br>- Escherichia coli<br>- Klebsiella pneumoniae<br>- Pseudomonas aeruginosa<br>- Proteus mirabili	4 µL		no	n.s.	70 – 3319	10000g for 5min	532nm<br>7mW<br>6-10sec	10 cm^{-1}	-	2h	-
114	J.A.M. Bispo	2013	Correlating the amount of urea, creatinine, and glucose in urine from patients with diabetes mellitus and hypertension with the risk of developing renal lesions by means of Raman spectroscopy and principal component analysis			SERS	PCA	n.s.	-	70	- Urea<br>- Creatinine<br>- Glucose	100 µL	-	no	-20°C	600 – 1800<br>(potentially 400-1800 $cm^{-1)}$	no	830 nm<br>300 mW<br>20s	n.s.	-	n.s.	Morning, fasting urine
115	K. Kinoshita	2012	Spectral pattern of urinary water as a biomarker of estrus in the giant panda			NIR	PLSR	n.s.	-	n.s.	Estrus	n.s.		no	-40°C	14700 – 4000	650g for 4 min	-	2 nm	n.s.	n.s.	-
116	Shapiro A.	2011	Raman Molecular Imaging: A Novel Spectroscopic Technique for Diagnosis of Bladder Cancer in Urine Specimens			RMI	PCA	n.s.	-	340	- Bladder Cancer<br>- voided urinary cells	n.s.	-	20 µm	n.s.	693 –1800<br>(potentially 350 – 3600)	2x<br>3000rpm for 5min	532 nm	n.s.	-	n.s.	-
117	G. Li	2011	Research on the Measurement of Urinary Albumin by Visible-Near Infrared Spectroscopy			NIR	PLS	n.s.	-	207	Albumin	n.s.	n.s.	n.s.	n.s.	n.s.	n.s.	n.s.	n.s.	n.s.	n.s.	Paper is only available in the Chinese language.
118	F. Zhai	2011	Rapid Determination of Ractopamine in Swine Urine Using Surface-Enhanced Raman Spectroscopy			SERS	- PLS<br>- PCA	n.s.	Gold-coated Klarite	240	Ractopamine	n.s.	0.4 µgmL^{-}	no	-20 °C	600 – 1800	5000rpm for 5min	780 n<br>10mW<br>20s	5 cm^{-1}	-	n.s	Samples kept frozen at -20 °C < 1 week
119	K. Kinoshita	2010	Near infrared spectroscopy of urine proves useful for estimating ovulation in giant panda (Ailuropoda melanoleuca)			NIR	- SIMCA	SPSS Version 10.0	-	53	Estrone-3-glucuronide (E1G)	n.s.	-	no	-40°C	4100 – 9100	650g for 4 min	-	8 cm^{-1}	n.s.	n.s.	Each sample was warmed up to 37 °C in water bath

							- Moving principal component analysis															
120	H. Wang	2010	Quantitative analysis of creatinine in urine by metalized nanostructured parylene			SERS	Linear correlation	n.s.	Ag-parylene	11	Creatinine	5 µL	0.5 µg/mL	no	−80 °C	400 – 1800	no	632.8 nm<br>35mW<br>10s	n.s	-	n.s.	The surfaces of SERS substrates were treated by a UV-Ozone cleaner for 2 min, then urine sample was added onto the substrate and let air dried
121	W. Liu	2008	Use of Artificial Neural Networks in Near-Infrared Spectroscopy Calibrations for Predicting Glucose Concentration in Urin			NIR	- PLS<br>- PCA<br>- Artificial neural network	n.s.	-	-	Glucose	A quartz cell with a path length of 1 mm	-	no	n.s.	4000 – 10000	n.s.	100W	2-4 cm^{-1}	3–4 times with distilled water	n.s.	-
122	K.W. Kho	2008	Polymer-based microfluidics with surface-enhanced Raman-spectroscopy-active periodic metal nanostructures for biofluid analysis			SERS	n.s.	n.s.	Au Colloid	1	Spectra enhancement	2ml	-	n.s	n.s	400 – 2000	n.s	632.8 nm<br>3.5mW<br>200s	n.s.	-	n.s.	-
123	D. Qi	2007	Chemical concentration measurement in blood serum and urine samples using liquid-core optical fiber Raman spectroscopy			LCOF-RS	- PLS<br>- Leave one out cross-validation<br>- Root-Mean-Squares Error of Cross-Validation	n.s.	-	61	- Urea<br>- Creatinine	3ml	-	no	4°C	800 – 1600	no	830 nm<br>160 mW<br>2s	7-13 cm^{-1}	-	n.s.	-
124	C.S. Park	2007	Classification of glucose concentration in diluted urine using the low-resolution Raman spectroscopy and kernel optimization methods			LRRS	- Kernel-based learning<br>- SVM<br>- PLS	n.s.	-	3	Glucose	n.s.	-	no	n.s.	200 – 2800	no	785nm<br>100mW<br>20s	15 cm^{-1}	-	n.s.	Spiked urine
125	Y.E. Kim	2006	Evaluation of Fourier transform near-infrared spectrometer for determination of oxalate in standard urinary solution			NIR-FT	- PCR<br>- PLSR<br>- MLR	n.s.	-	80	Oxalate	n.s.	10 mg/l	no	-	4000 – 12800	no	-	0.5 nm	n.s.	n.s.	Spiked urine
126	A.E. Guimaràes	2006	Near Infrared Raman Spectroscopy (NIRS): A technique for doping control			NIR-RS	Linear correlation	n.s.	-	5	Ephedrine	1 ml	4.8 µg/ml	no	-	700 – 1700	no	785nm<br>70 mW<br>100 s	5 cm^{-1}	n.s.	n.s.	Spectra obtained within one hour after collection
127	S. Tanaka	2006	Feasibility Study of a Urine Glucose Level Monitor for Home Healthcare Using Near Infrared Spectroscopy			NIR-FT	- PLS<br>- Leave-one-out cross validation method<br>- Standard Error of Calibration	Matlab	-	164	Glucose	n.s.	n.s.	no	n.s.	4000 – 9000	no	-	n.s.	n.s.	n.s.	-
128	C.S. Park	2006	Urine analysis in diluted situation using low-resolution Raman spectroscope			LRRS	- Data acquisition program using MFC<br>- PLS<br>- PCA<br>- RMSEP	Matlab R13	-	1	Glucose	200ml	5mg/dl	no	no	0 – 1800	no	785 nm<br>40mW	n.s.	-	n.s	-
129	T.L. Wang	2005	Semi-quantitative Surface Enhanced Raman Scattering Spectroscopic Creatinine Measurement in Human Urine Samples			SERS	Linear correlation	LabSpec	Au colloid	13	Creatinine	50 µl	2.56 mg/dl	no	4 °C	400 – 1700	no	632.8nm,<br>mW n.s.<br>20 s	1 cm^{-1}	-	n.s.	- Urine samples were taken out from the refrigerator and returned to 22°C.<br>- Spiked Urine<br>- Spectra obtained few hours after collection.
130	D. Qi	2005	Quantitative concentration measurements of creatinine dissolved in water and urine using Raman spectroscopy and a liquid core optical fiber			LCOF- RS	- PLS<br>- Leave-one-out cross validation	Matlab	-	42	Creatinine	1–3 mL for Cuvette/<br>200 µL fur LCOF		no	n.s.	600 – 1200	no	830nm<br>1s	n.s.	n.s.		Samples were stored in a refrigerator and used within 4 days
131	W. Liu	2005	Determination of Multiple Components in Urine Using FT-MIR, NIR and FT-Raman Spectroscopic Technique			FT-MIR, NIR, FT-RS	- PLS<br>- RMSEP	n.s.	-	n.s.	- Glucose<br>- Albumin	n.s.	n.s.	n.s.	n.s.	FTMIR 4000 – 400<br>NIR 370 – 15000<br>FTRS 300 – 4000		350mW	2 cm^{-1}	n.s.	n.s.	-
132	S.H. Chung	2005	Application of independent component analysis (ICA) method to the Raman spectra processing			RS	ICA by Maximum Likelihood (ML) fast fixed-point estimation algorithm	n.s.	-	18	- Glucose<br>- Creatinine<br>- Urea nitrogen<br>- Uric acid	n.s.	n.s.	no	-	50 – 2900	no	785nm<br>100mW	15 cm^{-1}	-	n.s.	?
133	P.A. Susmel	2004	Prediction of Purine Derivatives, Creatinine and Total Nitrogen Concentrations in Urine by FT-Near-Infrared Reflectance Spectroscopy (FT-NIR)			NIR-FT	PCR	Spectrum Quant+4.51	-	180	- Allantoin<br>- Uric acid<br>- Creatinine<br>- Total Nitrogen	100 µl	0.048 g/l<br>0.011g/l<br>0.06 g/l<br>0.94 g/l	Yes (filter paper)	n.s.*	4000 – 7000	no	-	2 cm^{-1}	n.s.	<30sec	Samples were analysed fresh or after be frozen for a few days
134	W.R. Li	2003	Direct spectrometric determination of proteins in body fluids using a near-infrared cyanine dye			NIR	Recovery test	n.s.	-	n.s.	Total proteins	0.05 mL	20 ngmL–1	no	n.s.	11100 – 20000	no	-	n.s.	-	n.s.	-

135	J.W. McMurdy	2003	Raman Spectroscopy-Based Creatinine Measurement in Urine Samples from a Multipatient Population	RS	- Hybrid linear analysis calibration<br>- PLS<br>- Leave-one-out cross-validation<br>- RMSECV	n.s.	-	19	Creatinine	n.s	24 mg/dL	no	n.s.	500 – 1800	no	830nm<br>200 mW<br>900 s	5 cm^{-1}	-	n.s.	Samples were kept at 7°C and measured within few days
136	S. Cystron	2003	Fiberoptic infrared spectroscopy: a Novel tool for the analysis of urine and urinary salts in situ in real time	FTIR	Calibration curves	n.s.	-	48	Salts	5mL	Phosphate 5mmol/L; Carbonate 8mmol/L; Oxalate 6mmol/L; Urate 3mmol/L.	no	no	900 – 1700<br>(potentially 800 – 4000)	no	-	4 cm^{-1}	n.s.		-
137	R. Somorjai	2002	Distinguishing normal from rejecting renal allografts: application of a three Stage classification strategy to MR and IR spectra of urine	FTIR	- Feature space reduction<br>- Optimal region selector<br>- Genetic algoritms	n.s.	-	68	Rejecting renal allografts	12 ml	-	no	n.s.	800 – 5000	no	-	4 cm^{-1}	n.s.	n.s.	-
138	L. Pezzaniti	2001	Preliminary investigation of near-infrared spectroscopic measurements of urea, creatinine, glucose, protein, and ketone in urine	NIR	PLS	Matlab	-	120	- Urea<br>- Creatinine,<br>- Glucose<br>- Protein<br>- Ketone	n.s.	n.s.	0.45 µm	no	4000 – 9000	no	-	2 cm^{-1}	-	n.s.	-
139	W. Premasiri	2001	Urine analysis by laser Raman spectroscopy	RS/SERS	Calibration curve	n.s.	Gold Colloidal	5	- Urea<br>- Uric acid<br>- Creatinine	50 µL		no	no	400 – 1900	no	785nm<br>120mW<br>20s	n.s.	n.s.	n.s.	- No Storage<br>- Room temperature analyse<br>- Human and artificial urine
140	H.M. Heise	2001	Multivariate Calibration for the Determination of Analytes in Urine Using Mid-Infrared Attenuated Total Reflection Spectroscopy	ATR-FTIR	PLS	Matlab	-	67	- Urea<br>- Creatinine<br>- Uric acid<br>- Glucose<br>- Total protein,<br>- Phosphate<br>- Sulfate	50 µL	-	no	-35 °C	1800 – 800	no	-	4 cm^{-1}	1% aqueous solution of Dextran enzymatic from Merck and ethanol	2min	Analyse at 30°C
141	R.A. Shaw	2000	Toward Reagent-free Clinical Analysis: Quantitation of Urine Urea, Creatinine, and Total Protein from the Mid-Infrared Spectra of Dried Urine Films	FTIR	PLS	n.s.	-	200	- Urea<br>- Creatinine<br>- Total Protein	12 µL	n.s.	no	n.s.	900 – 1500<br>(potentially 800-5000)	no	-	4 cm^{-1}	n.s.	n.s.	Ambient temperature dried sample
142	H.M. Heise	1999	Quantitation of Metabolites and Salts in Urine by Attenuated Total Refection Infrared Spectroscopy, in Fourier Transform Spectroscopy	ART-FTIR	PLS	Matlab	-	67	- Urea<br>- Creatinine<br>- Uric acid<br>- Phosphate<br>- Solphat<br>- Glucose	50 µL	n.s.	no	-35 °C	400 – 4000	no	-	4 cm^{-1}	A cleaning cycle using a detergent (1% aqueous solution of Dextran enzymatic from Merck, Darmstadt), distilled water, and ethanol	n.s.	Cell temperature was mamtained at 30°C
143	X. Dou	1997	A highly sensitive, compact Raman system without a spectrometer for quantitative analysis of biological samples	FT-RS	Correlation coefficient	n.s.	-	-	- Glucose<br>- Acetone	500 µL	n.s.	no	no	300 – 1900	no	980nm<br>100mW<br>1s	n.s	-	n.s.	- Spiked Urine<br>- Samples analysed within 1 Hour
144	R.A. Shaw	1996	Quantitation of protein, creatinine, and urea in urine by near-infrared spectroscopy	NIR	- Multiple wavelength linear regression<br>- PLS	n.s.	-	177	- Protein<br>- Creatinine<br>- Urea	n.s.	n.s.	no	n.s.	400 – 2400	no	-	n.s	n.s.	n.s.	-
145	X. Dou	1996	Quantitative Analysis of Metabolites in Urine by Anti-Stokes Raman Spectroscopy	RS	Correlation coefficient	n.s.	-	3	- Glucose<br>- Acetone<br>- Urea	n.s.	Gluc. 120 mg/dL<br>Ace. 6.58 mg/ml<br>Ur. 35.6 mg/ml	no	n.s.	200 – 2500	no	514nm<br>100mW<br>1-5s	6 cm^{-1}	n.s.	n.s.	Spiked Urine
146	X. Dou	1996	Quantitative analysis of metabolites in urine using a highly precise, compact near-infrared Raman spectrometer	NIR-RS	Correlation coefficient	n.s.		n.s.	- Glucose<br>- Acetone<br>- Urea<br>- Creatinine	n.s.		no	n.s.	0 – 2500	no	820nm<br>130mW<br>1s	2 cm^{-1}	n.s.	n.s.	-
147	A.W. van Toorenenbergen	1994	Assay of glucose in urine by near-infrared spectrophotometry	NIR	Multiple linear regression	Quattro pro 4.0	-	52	Glucose	n.s.	n.s.	no	n.s.	1440 – 2360	no	-	n.s.	n.s.	<1min	-

148	K.S. Kalasinskyt	1993	Detection of Amphetamine and Methamphetamine in Urine by Gas Chromatography/Fourier Transform Infrared (GC/FTIR) Spectroscopy		GC/FTIR	coefficient of variation	n.s.	-	n.s.	- Amphetamine<br>- Methamphetamine	2 µL	10 ng/mL.	n.s.	n.s.	500 – 4000 cm^{-1}	n.s.	-	8 cm^{-1}	n.s.	n.s.	-Spiked Urine
149	J.T. Grismer	1966	Infrared Spectroscopy and Osmolality Analysis of Urine: Two Simple Sensitive Methods for Early Detection of Postoperative Anuria After Thoracotomy		IR	n.s.	n.s.	-	30	- Osmolality<br>- Urea<br>- Sulfates<br>- mono- and di-basic Phosphates<br>- Gucose<br>- Dextran	0.5 ml	Urea 200mg /mL	no	n.s.	1000 – 1550	no	-	n.s.	n.s.	12min	Urine sample collected prior to the surgical procedure, through catheter
150	L.T. Rozelle	1965	Identification of Major Infrared Absorbing Components of Human Urin		IR	n.s.	n.s.	-	n.s.	- Phosphate<br>- Solphate<br>- Urea<br>- Glucose	n.s.	-	no	< 0 °C	1000-1500	no	-	n.s.	The sample cells were cleaned with distilled water after use and flushed with appropriate Urine sample	n.s.	The urine samples were allowed to come room temperature before analyse Analyze at 35°C
151	J. Clausen	1963	Mucopolysaccharidosis: Paper Electrophoretic and Infra-Red Analysis of the Urine in Gargoylism and Morquio-Ullrich's Disease		IR	n.s.	n.s.	-	n.s.	Mucopolysaccharides	n.s.	n.s.	yes<br>Whatman n. 1 filter	n.s.	650 – 5000	n.s.	n.s.	n.s.	n.s.	n.s.	-
152	K. Dobriner	1948	The application of infrared spectrometry to fractionation of urinary metabolism ketosteroids		IR	Calibration curve	n.s.	-	-	Ketosteroids	0.2ml	0.5%solution	no	n.s.	800 – 1200	n.s.	n.s.	n.s.	Carbon disulfide	<10min	-

Colours-Legend

(light blue)	Method or Technique without in Test in-Vivo or in Clinical setting
(light yellow)	Method or Technique applied for Oncological purpose
(light green)	Method or Technique applied in Clinical /In Vivo setting- non-Oncological
(light red)	Animal Urine
(pink)	Artificial Urine

Abbreviations

AuNPs	Gold nanoparticles	GA	Genetic Algoritms
AuNRs	Gold Nonorods	Au/ZnO NRS	Zink Oxid Gold Nanorods
PCA	Principal Component Analysis	AgNPs	Silver nanoparticles
SIMCA	soft independent modelling of class analogy	PLS	Partial least squires
SNV	Standard Normal Variate	LDA	Linear discriminant analysis
MCS-ALS	Multivariate curve resolution alternating least square	MLR	Multilinear regression
AgNF	nanoporous silver film	CLS	classic least squares
AgNCs	silver nanocubes	SEP	Standard error of prediction
MCR-ALS	multivariate curve resolution-alternating least squares	DFA	discriminant function analysis
PLSR	Partial least squares regression	RMSEC	root mean square error of calibration
GNPs	colloidal gold nanoparticles	RMSECV	root mean square error of cross validation
GA-PLS-LDA	Genetic algorithms-partial least squares-linear discriminant analysis	RMSEP	root mean square error of prediction
SiO2–AuNPs	Silicon chips with a self-assembled monolayer of Gold nanoparticles	PCR	principal component regression
RSD	Relative Standard deviation	MPCA	Moving principal component analysis
