# Supplementary material for: Vibrational Spectroscopy in Urine Samples as a Medical Tool: Review and Overview on the Current State-of-the-Art
Source: Diagnostics (Basel). 2022 Dec 22;13(1):27. doi: 10.3390/diagnostics13010027 (PMC9818072; doi:10.3390/diagnostics13010027)
Supplement: Supplementary file 1 [file diagnostics-13-00027-s001.zip › Supplemental materials-Table S2 - Review papers .pdf]

Table S2: Supplementary Materials; Review Papers. F.Vigo et al., 2022, Diagnostics.

Autor		Year	Title	Technique
1	*B. Formoso*	2020	Cancer Diagnosis through SERS and Other Related Techniques	SERS
2	*K. Naseer*	2020	ATR-FTIR spectroscopy as the future of diagnostics: a systematic review of the approach using bio-fluids	ATR-FTRI
3	*Y. Borong*	2019	Development of surface-enhanced Raman spectroscopy application for determination of illicit drugs: Towards a practical sensor	SERS
4	*S. Pahlow*	2018	Application of Vibrational Spectroscopy and Imaging to Point-of-Care Medicine: A Review	Vibrational Spectroscopy
5	*L.B. Leala*	2018	Vibration spectroscopy and body biofluids: Literature review for clinical applications	Vibrational Spectroscopy
6	*S. Pahlow*	2018	Application of Vibrational Spectroscopy and Imaging to Point-of-Care Medicine: A Review	Vibrational Spectroscopy
7	*Z. Xiao-Shan*	2018	Label-free SERS in biological and biomedical applications: Recent progress, current challenges and opportunities	SERS
8	*J. Smolsky*	2017	Surface-Enhanced Raman Scattering-Based Immunoassay Technologies for Detection of Disease Biomarkers	SERS
9	*W. Christian*	2016	Critical Review Upon the Role and Potential of Fluorescence and Near-Infrared Imaging and Absorption Spectroscopy in Cancer Related Cells, Serum, Saliva, Urine and Tissue Analysis	IR
10	*M.L. Baker*	2016	Developing and understanding biofluid vibrational spectroscopy: a critical review	Vibrational Spectroscopy
11	*A. Bonifacio*	2015	Label-free surface-enhanced Raman spectroscopy of biofluids: fundamental aspects and diagnostic applications	SERS
12	*U. Neugebauer*	2015	Raman spectroscopy towards clinical application: drug monitoring and pathogen identification	RS
13	*A.L. Mitchell*	2014	Vibrational spectroscopy of biofluids for disease screening or diagnosis: translation from the laboratory to a clinical setting	Vibrational Spectroscopy
14	*E. Mauriz*	2020	Low-Fouling Substrates for Plasmonic Sensing of Circulating Biomarkers in Biological Fluids (SERS)	SERS
15	*G. Hoşafçı*	2007	Clinical chemistry without reagents? An infrared spectroscopic technique for determination of clinically relevant constituents of body fluids	IR
16	*R. Meier*	2005	Vibrational spectroscopy: a 'vanishing' discipline?	Vibrational Spectroscopy
17	*M. Janelle*	2005	Photodiagnosis using Raman and surfaceenhanced Raman scattering of bodily fluids	SERS

ATR-FTRI: Attenuated Total Reflection Fourier Transform Infrared Spectroscopy; SERS: Surface-Enhanced Raman Spectroscopy; IR: Infrared Spectroscopy
